# Design and experimental analysis of dual-band polarization converting metasurface for microwave applications

**DOI:** 10.1038/s41598-020-71959-y

**Published:** 2020-09-21

**Authors:** Bilawal Khan, Babar Kamal, Sadiq Ullah, Imran Khan, Jawad Ali Shah, Jingdong Chen

**Affiliations:** 1 Department of Telecommunication Engineering, UET Mardan, Mardan, 23200 Pakistan; 2 Center of Intelligent Acoustics and Immersive Communications, Northwestern Polytechnical University, Xi’an, Shaanxi China; 3 Department of Electrical Engineering, UET Mardan, Mardan, 23200 Pakistan; 4 Electronic Technology, Universiti Kuala Lumpur, British Malaysian Institute, Selangor, Malaysia

**Keywords:** Engineering, Materials science, Physics

## Abstract

The manipulation of polarization state of electromagnetic waves is of great importance in many practical applications. In this paper, the reflection characteristics of a thin and dual-band metasurface are examined in the microwave frequency regime. The metasurface consists of a 22 × 22 element array of periodic unit cells. The geometry of the unit cell consists of three layers, including a 45° inclined dipole shape metal patch on top, which is backed by a 1.6 mm thick FR-4 substrate in the middle, and a fully reflective metallic mirror at the bottom. The proposed surface is exposed to horizontally (*x*) or vertically (*y*) polarized plane waves and the co and cross polarization reflection coefficients of the reflected waves are investigated experimentally in the 6–26 GHz frequency range. The metasurface is designed to convert incident waves of known polarization state (horizontal or vertical) to orthogonal polarization state (vertical and horizontal) in two distinct frequency bands, i.e. 7.1–8 GHz and 13.3–25.8 GHz. In these two frequency bands the simulated and experimental results are in good agreement. The polarization conversion ratio (PCR) of the surface is greater than 95% in the targeted frequency bands. A detailed parametric analysis of the metasurface is also discussed in this work and it has been estimated that the surface has the additional ability to convert linearly polarized waves to circularly polarized waves at several distinct frequencies. The proposed metasurface can be utilized in sensor applications, stealth technology, electromagnetic measurements, and antennas design.

## Introduction

Metasurfaces are widely used for polarization control and wave-front shaping of electromagnetic waves in the microwave and optical frequency bands. Polarization is the alignment of electric field component of an electromagnetic wave in space. The manipulation of the polarization of electromagnetic wave in the microwave-to-optical frequency range, has received a great attention, from researchers around the globe due to its diverse applications^1,2^. Applications of polarization manipulative metsurfaces includes, optical vertex converters^2^, optical metrology^3^, quarter^4^ and half-wave plates^5^, polarimetry^6^, planar lenses^7^, GPR detection^8^, multi spectral photography^9^, biological sensing^10^ and antennas^11^. Polarization can be manipulated either through conventional methods of using natural materials^12^ or through artificially engineered materials. Faraday Effect, the optical activity of crystals, proteins with helical secondary structure, gases or solutions of chiral molecules (sugar), and chiral liquid crystals are few of the conventional techniques^12^ to control polarization. However, the mentioned converters have the limitation of narrowband frequency response as well as thicker and bulky volume, due to which these converters are inappropriate for use within ultra-thin device sensors and nanophotonic devices. Consequently, artificially engineered materials have been developed for wideband and miniaturized polarization control devices. The most prominent and artificially man-made materials designed for diverse range of microwave and optical applications are known as Metamaterials (MMs)^13,14^. These materials acquired bigger attention because of their fascinating applications such as invisible cloaks^15,16^, negative refraction^17,18^, super diffraction imaging^19,20^, fano-resonance^21^, multi-functional reflectors^22^, transformation optics^23^, beam manipulation^24,25^ and flat lens^26^, to name but a few.

Metasurfaces^27–30^ are the two-dimensional equivalent of metamaterials, having the advantage of relaxed fabrication complexity. These metasurfaces can be designed and characterized in two distinct modes, i.e. transmission and reflection. A flexible and ultra-thin metasurface (MS) consisting of a single cross placed in a split ring resonator (SRR)^31^ was used for transmission purposes. The surface was able to achieve half-mirror and quarter-wave plate operation in the microwave region. Similarly, a bi-layer anisotropic metasurface working as a half and quarter wave plate^32^ was designed using a double SRR on both sides of the substrate with a concentric metallic loop inside. In transmission-mode, higher transmission efficiency can be achieved if the metsurface is thin^33^. Higher efficiency can be attained if the surface is operated in the reflection mode due to the total reflection of the incoming wave. Recently, several metasurfaces working in the reflection mode have been reported in the literature for polarization conversion in the microwave^34–38^ and terahertz^39^ frequency ranges. Two SRRs were proposed on a sub wavelength unit cell structure for multi plasmonic^34^ resonance in the microwave frequency region. Another anchor shaped metasurface^38^ was designed using genetic optimization algorithm, which give ultra-wideband polarization conversion bandwidth in five plasmonic resonances. Despite of the many advantages like excellent polarization conversion efficiency, sub-wavelength thickness, wide bandwidth, there is still an issue with polarization converting metasurface; that is dual band, i.e., to convert incident waves of known polarization state to orthogonal polarization state in the microwave frequency range.

In this article, a unique dipole shaped metasurface is realized in the microwave frequency regime, which gives conversion of *x*-polarized waves to *y*-polarized waves and vice versa in two distinct frequency bands. It is also estimated that the proposed metasurface has the ability to give linear-to-circular polarization conversion at four distinct frequencies.

## Structure design and related theory

**Figure 1:**
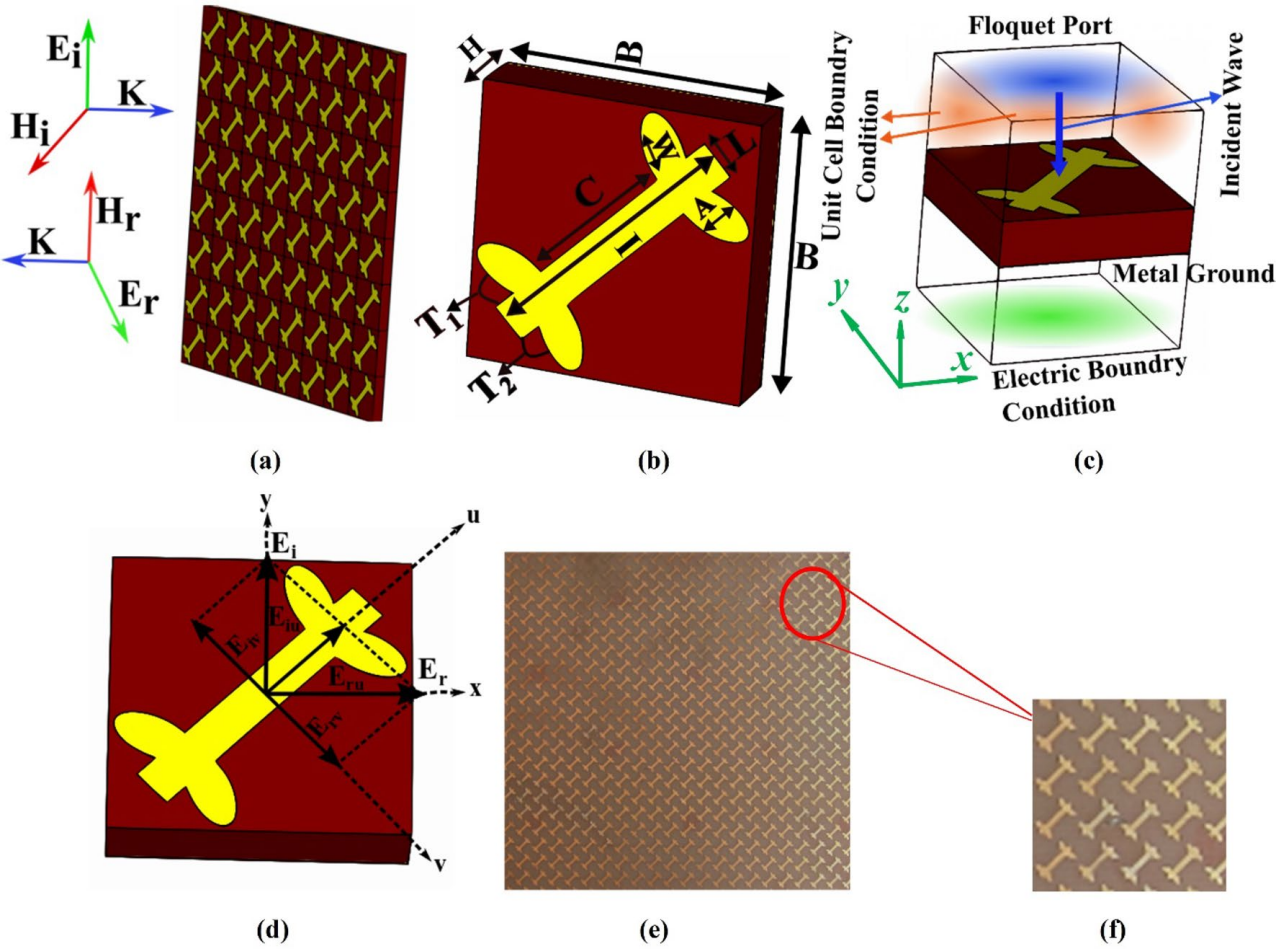
The proposed metasurface: **(a)** schematic diagram, **(b)** angled view of the unit cell, **(c)** simulation setup **(d)** top view of the unit cell along *u* and *v* coordinate system, **(e)** picture of the fabricated metasurface, **(f)** zoomed view of the surface.

Design of the proposed polarization converting metasurface is shown in Fig. 1a. The upper layer of the unit cell of the metasurface is a leaf-shaped dipole made of copper with a conductivity of 5.8 × 10^7^S/m. The middle layer is FR-4 substrate having dielectric constant, loss tangent, and thickness of 4.4, 0.02 and 1.6 mm, respectively. The bottom layer is a fully reflective copper ground plane. The thickness of the copper patch used in both top and bottom layers is 0.018 mm. The remaining dimensions of the unit cell are labelled in Fig. 1b: i.e., *B* = 7, *I* = 7, *A* = 0.5, *W* = 1, C = 4.4, *L* = 1 mm. The angle between the major axis of each elliptical leaf and the dipole is T1 = T2 = 20 degrees.

The structure is analyzed and optimized using the Finite Difference Time Domain (FDTD) approach employed in CST Microwave Studio. Periodic boundary conditions have been applied along the *x* and *y* directions to simulate and characterize scattering parameters of the periodic array of the unit cell. The structure has been illuminated with linear (*x* and *y*) polarized plane waves by assigning a Floquet port at the top of the unit cell. The simulation setup is shown in Fig. 1c. The generalized mathematical representation of the aforementioned *x* and *y* polarized incident waves are $E_{xi} = E_{xi} e_{x } {\text{and}} E_{yi} = E_{yi} e_{y}$, where ($e_{x }$, $e_{y}$) are the unit vectors along the *x* and *y* directions, respectively, and the subscript ‘*i*’ is used for ‘incident’ waves. For a *y*-polarized incident wave, the reflected waves can be expressed as $E_{r} = E_{xr} e_{x} + E_{yr} e_{x} = R_{xy} \exp \left( {j\emptyset_{xy} } \right)E_{yi} e_{x} + R_{yy} \exp \left( {j\emptyset_{yy} } \right)E_{yi} e_{y}$; where $R_{xy} = \left| {{{E_{xr} } \mathord{\left/ {\vphantom {{E_{xr} } {E_{yi} }}} \right. \kern-\nulldelimiterspace} {E_{yi} }}} \right|$ and $R_{yy} = \left| {{{E_{yr} } \mathord{\left/ {\vphantom {{E_{yr} } {E_{yi} }}} \right. \kern-\nulldelimiterspace} {E_{yi} }}} \right|$, are the cross and co-polarized reflection coefficients, respectively. The former (i.e. $R_{xy}$) gives the fraction of *x*-polarized component of the reflected wave. The latter (i.e. $R_{yy}$), gives the fraction of the *y*-polarized component of the reflected wave. The phase angles of the co and cross polarization reflection coefficients are represented by $\emptyset_{yy}$ and $\emptyset_{xy}$, respectively. If an anisotropy is introduced in the unit cell of the metasurface, the relative magnitude and phase of the reflected wave ($E_{xr}$ and $E_{yr}$) may be different^40^. Similarly the possibility of conversion of the incident wave from linear to circular wave can occur under special circumstances, i.e., if $R_{xy} = R_{yy}$ and $\Delta \emptyset = \emptyset_{yy} - \emptyset_{xy} = \pm 90^{ \circ } = 2n\pi \pm \pi /2$ , where *n* is an integer. Depending on the frequency, $\Delta \emptyset$ can take values within the range of − 180° to 180°, showing all possible conversion states (i.e., linear, elliptical and circular.) for the waves upon reflection^40,41^.

To better comprehend the polarization conversion property of the proposed metasurface, we analyze another parameter called polarization conversion ratio (PCR), which has been used in the literature^42–45^. The PCR is the ratio of square of the cross-polarization reflection coefficient to the sum of the square of the co and cross polarization reflection coefficients. For *x*-polarization incidence, cross and co-polarized reflection coefficients are $R_{yx} = \frac{{E_{yr} }}{{E_{xi} }}$ and $R_{xx} = \frac{{E_{xr} }}{{E_{xi} }}$, whereas the corresponding coefficients for the *y*-polarization incident waves are $R_{xy} = \frac{{E_{xr} }}{{E_{yi} }}$ and $R_{yy} = \frac{{E_{yr} }}{{E_{yi} }}$, respectively; where the subscripts “*i*” and “*r*” represent incident and reflected waves. The PCR can be deduced as follows^35^:

1 $$PCR = \frac{{\left| {R_{yx} } \right|^{2} }}{{\left| {R_{yx} } \right|^{2} + \left| {R_{xx} } \right|^{2} }} = \frac{{\left| {R_{xy} } \right|^{2} }}{{\left| {R_{xy} } \right|^{2} + \left| {R_{yy} } \right|^{2} }}$$

## Results and discussion

### Frequency response of the reflection coefficient

**Figure 2:**
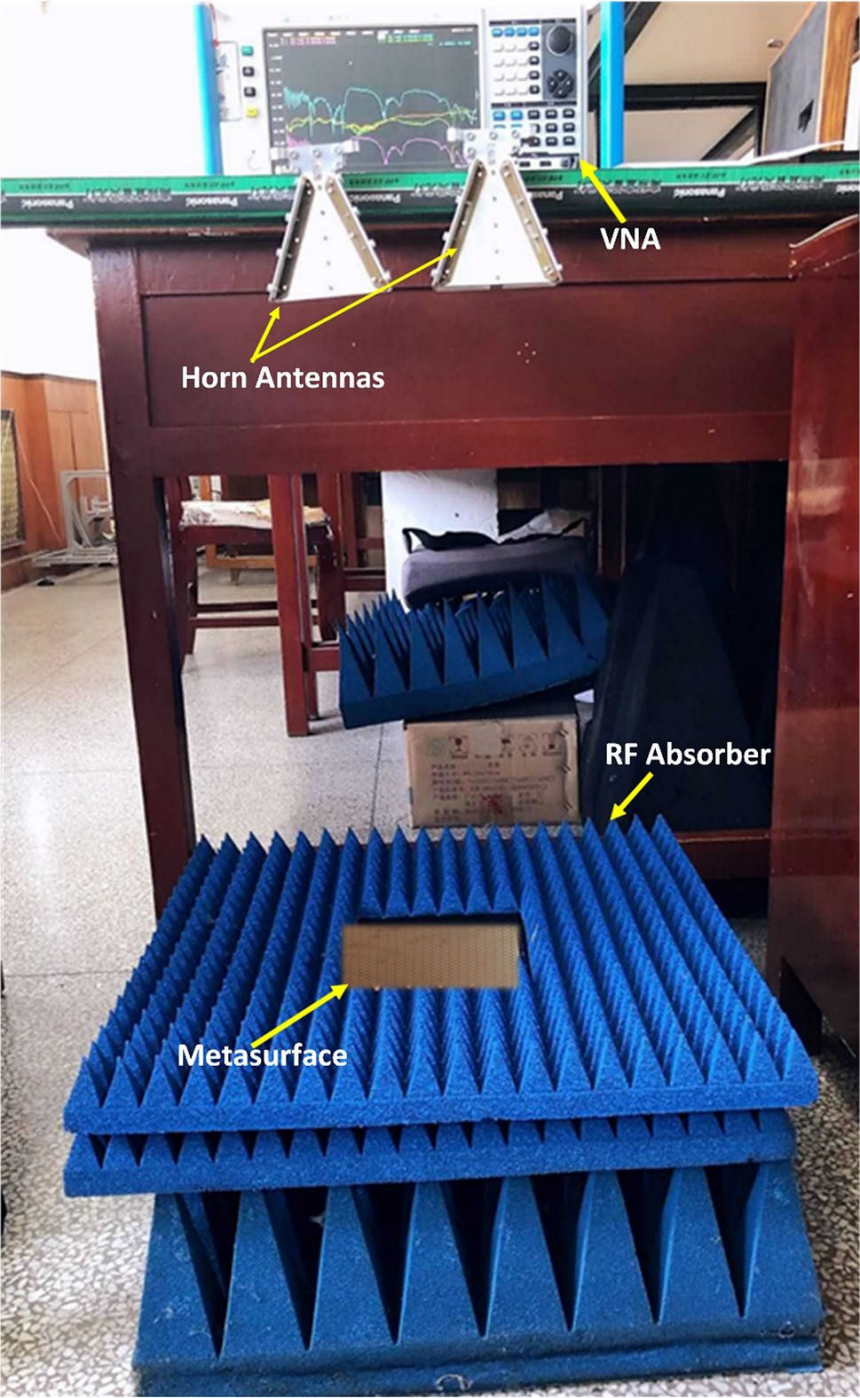
Experimental setup.

For experimental validation, the proposed 154*154 mm^2^ metasurface was fabricated on FR-4 substrate, backed by metallic (copper) ground plane. The number of unit cells accommodated within the area of the fabricated metasurface are 424 (= 22 × 22). The measurement is conducted using the setup shown in Fig. 2. The co and cross polarization reflection coefficients are measured using two ridged broadband (18–26 GHz) horn antennas, both connected to the Agilent N5232A vector network analyzer (VNA). One horn act as a transmitting antenna while the other works as a receiving antenna. One horn antenna irradiates the metasurface while the other is used to receive the reflected wave from the metasurface. The co-polarization reflection coefficients (*R*_*xx*_, *R*_*yy*_) are measured by positioning both horn antennas along horizontal or vertical orientation, respectively. To measure the cross-polarization reflection coefficient (*R*_*yx*_), the receiving antenna was placed in vertical state while the transmitting horn antenna was horizontally positioned. However, for measuring *R*_*xy*_, the transmitting and receiving horn antennas were employed in vertical and horizontal positions, respectively.

The setup was carefully calibrated and the measurements of the metasurface under test were carried out with reference to a metallic (copper) surface. Using the measurement setup in Fig. 2, the radar cross section (*RCS*) of the metasurface ($\sigma_{MS}$) and the reference copper surface ($\sigma_{Cu}$) were measured for an incident wave of known polarization (i.e. *x* or *y*). The measured reflection coefficients were extracted by normalizing the measured RCS of the metasurface, relative to the RCS of the reference copper surface. The following expressions were used in the evaluation of the measured reflection coefficients:

2 $$\sigma_{MS} = 4\pi \rho^{2} \frac{{S_{r} }}{{S_{i} }}$$

where *S*_*r*_ and *S*_*i*_ are the power density of the scattered and incident waves, respectively in Wm^−2^. $\rho$, is the distance between the horn antennas and the surface under the test. The power density of the scattered and incident waves from the reference copper surface was equal and hence the ratio of *S*_*r*_ to *S*_*i*_ is unity and hence the *RCS* of the copper surface is given by:

3 $$\sigma_{Cu} = 4\pi \rho^{2} \left( 1 \right)$$

The reflection coefficient for a given state of polarization was evaluated by normalizing the *RCS* of the metasurface relative to that of the metallic surface, i.e.

4 $$R_{ij} = \frac{{\sigma_{MS} }}{{\sigma_{Cu} }}\;\;\;i,j = x \;or\; y$$

**Figure 3:**
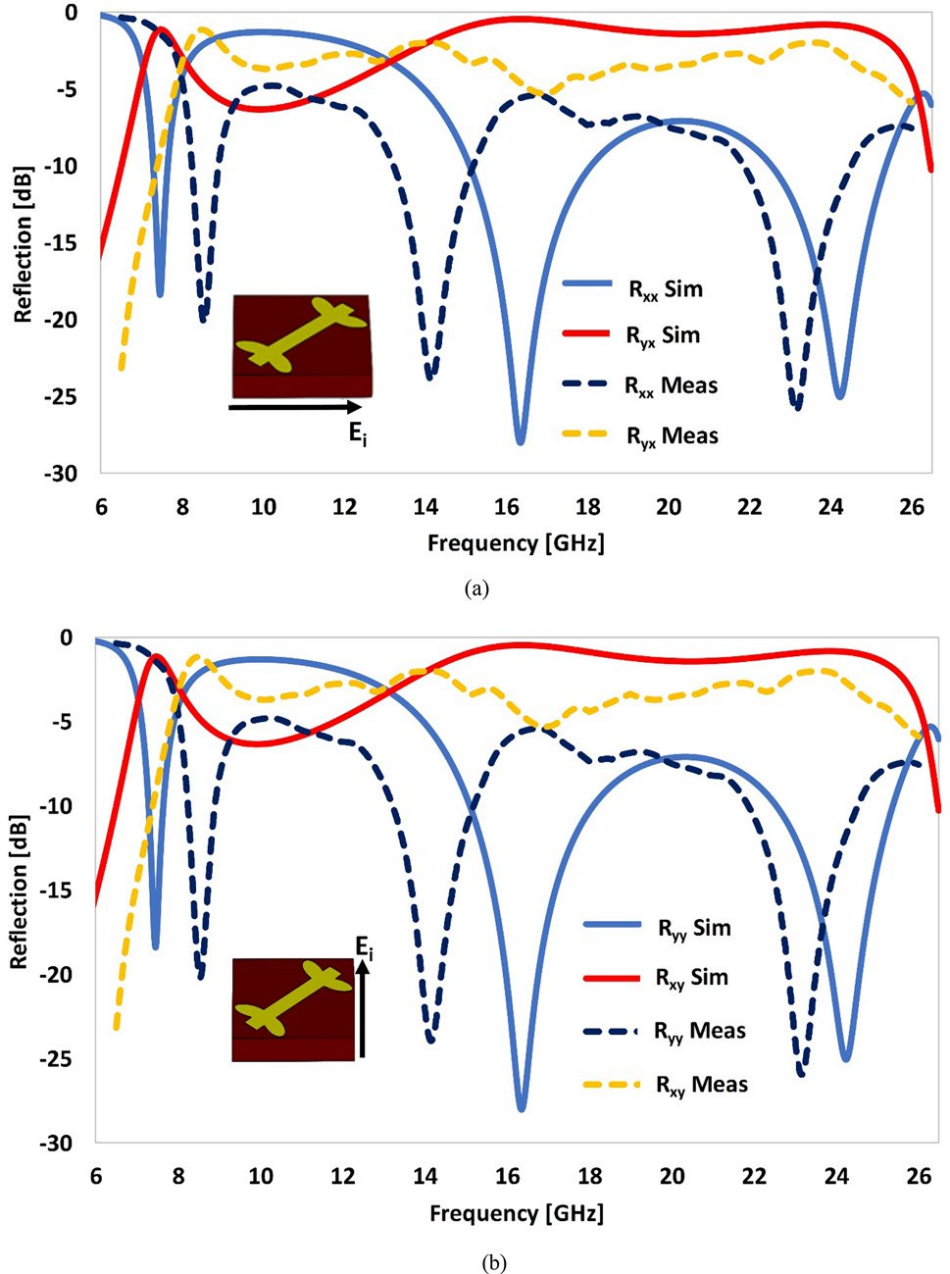
Comparison of the simulated and measured reflection coefficients: **(a)** *x*-polarized wave incidence. **(b)** *y*-polarized wave incidence.

Figure 3a,b illustrate the comparison of the simulated and measured reflection coefficients for *x* and *y* polarized wave incidence, respectively. Based on simulated and experimental results, the engineered structure can function as a dual-band polarization conversion device in two distinct frequency bands: 7–8 GHz and 13–25 GHz. The co-polarization reflection coefficients (*R*_*xx*_ and *R*_*yy*_) are less than − 10 dB in the two frequency bands, which shows that the reflected waves do not retain the original polarization state of the incident waves. On the other hand, the cross-polarization reflection coefficients (*R*_*xy*_ and *R*_*yx*_) are predominantly higher (≥ − 3 dB) than their co-polarization counterparts in these frequency bands, which confirms that the polarization state of the incident waves has been transformed into the orthogonal polarization state, after reflection from the proposed metasurface. Except minor differences, the experimental and simulated results are in good agreement. The slight variance in the two is mainly due to the default periodic boundary conditions used in simulations, where an infinite repetition of the unit cells along *x* and *y* directions is assumed, which is impossible in the real environment, where we have actually tested a prototype of finite size (22 × 22 unit cells). The finite size of the structure leads to edge diffraction, which results in a slight deviation between the simulated and experimental results. Other reasons that may cause deviation include fabrication tolerances, calibration and human errors involved in fixing the relative angle between the metasurface, transmitting, and receiving antennas.

**Figure 4:**
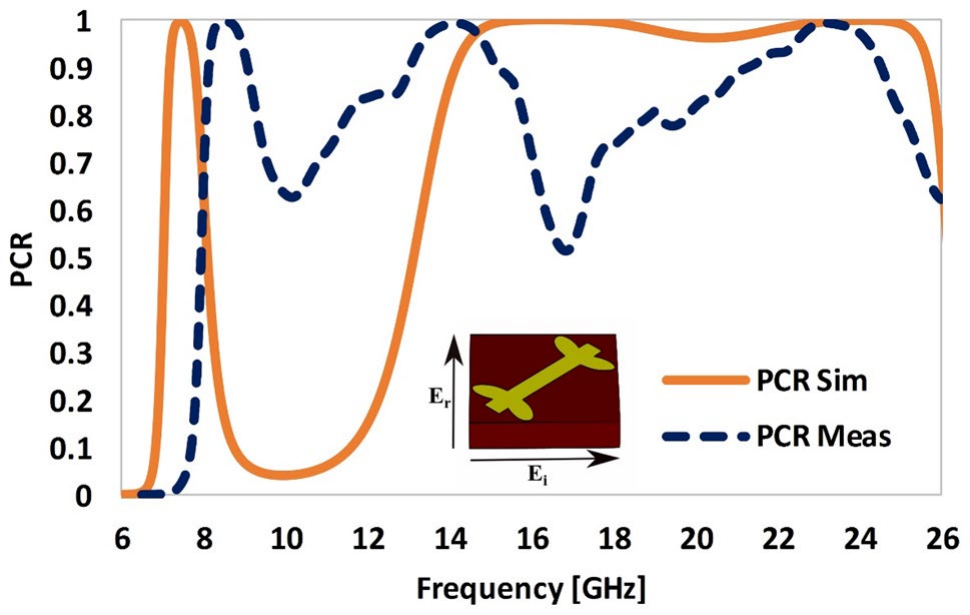
Polarization conversion ratio (PCR).

The phenomenon of polarization conversion is better illustrated by the PCR of the proposed metasurface, which shows the two prominent resonances with excellent measured PCR (> 95%) in the first resonant band and a PCR ~ 60–95% in the second resonant band. The first resonance occurs at 7.4 GHz while the second wideband resonance covers the 13.5 to 25.7 GHz frequency range. In these frequency ranges a linearly polarized incident wave of known polarization state (*x* or *y*) is converted to the orthogonally polarized reflected wave (*y* or *x*), respectively (Fig. 4). The main reason for the reduction of PCR in the higher resonant band is the lossy nature of the FR-4 substrate at higher frequencies. However, if low loss substrates are used the losses at higher frequencies can be minimized.

### Surface current distribution

**Figure 5:**
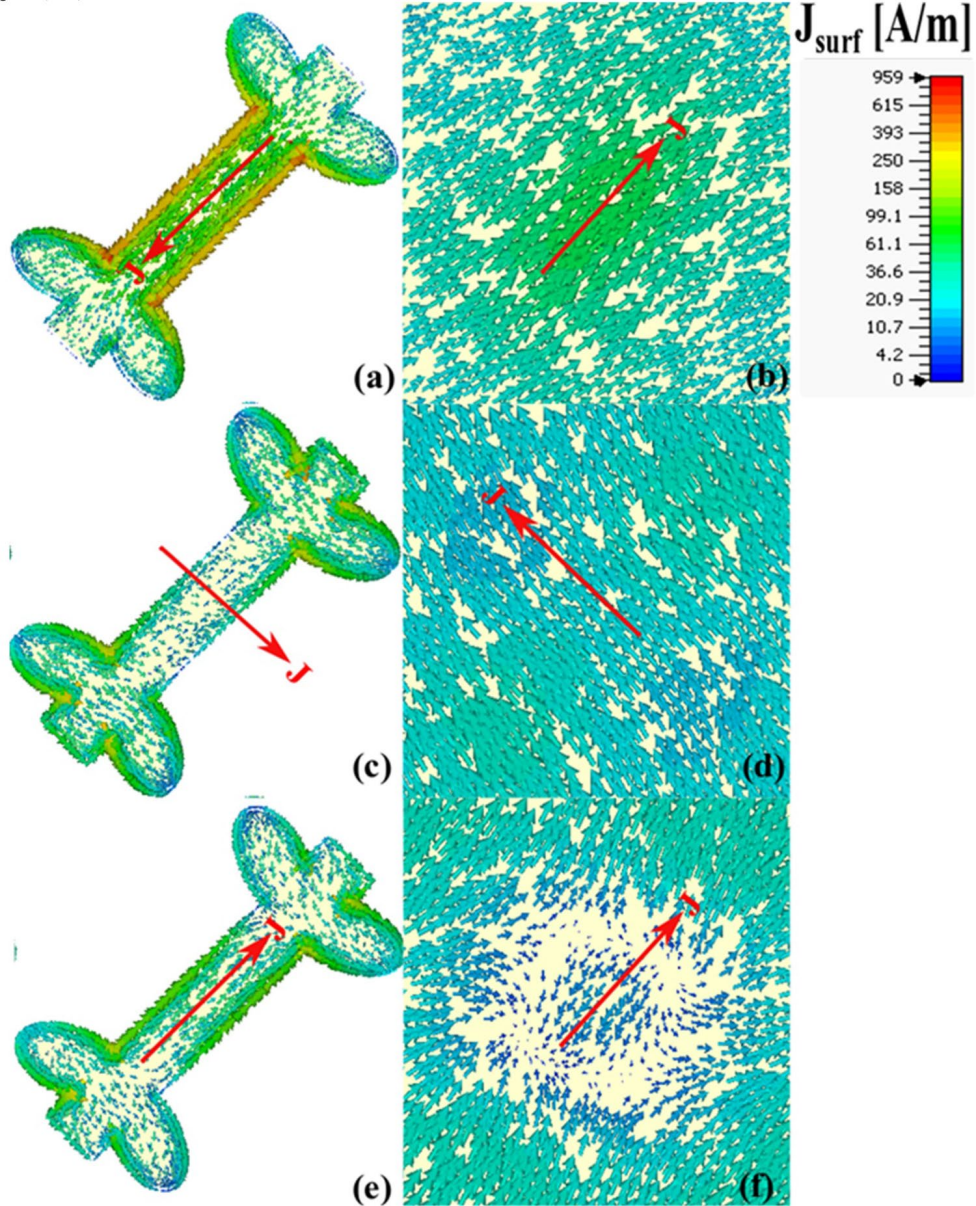
Surface current distribution: **(a)** patch at 7.4 GHz, **(b)** ground at 7.4 GHz, **(c)** patch at 16.4 GHz, **(d)** ground at 16.4 GHz, **(e)** patch at 24.2 GHz, **(f)** ground at 24.2 GHz.

The distribution of surface current density on the patch and ground indicate the cause of electric or magnetic resonance. For an electric dipole resonance, the distribution of current density on the surface of patch should be in phase (parallel) to the distribution of current on the ground. But when the distribution of current density on the patch is out of phase (anti-parallel) to the distribution of current density on the ground then magnetic resonance will occur. To give more physical insight into the polarization conversion ability of the proposed metasurface, the distribution of surface current density is demonstrated at the attained resonant frequencies (Fig. 5). In Fig. 5 the net surface current density is denoted by ‘*J’* while the head of the arrow indicate the direction of the current on the surface. The current distribution on the top layer (patch) and the bottom layer (ground) of the metasurface are antiparallel, which generates plasmon/magnetic resonance (or magnetization) in the dielectric substrate through the current loops^34^. Hence magnetic resonance occurs at *f*_*1*_ = 7.4 GHz (Fig. 5a,b), *f*_*2*_ = 16.4 GHz (Fig. 5c,d). It is worth noticing that the distribution of the surface current density of patch and ground at 24.2 GHz is in-phase (parallel) to each other. It indicates an electric dipole resonance at 24. 2 GHz (Fig. 5e,f).

### Parametric analysis

The characteristics of the dipole shaped metasurface are extensively studied in the literature^38,39,44^. All the plasmonic resonance^34^ are caused by electric and magnetic response of the metasurface to the incident electromagnetic waves. This section covers the parametric analysis for designing the metasurface of Fig. 1.

### Optimization of the structural geometry of the top dipole structure

**Figure 6:**
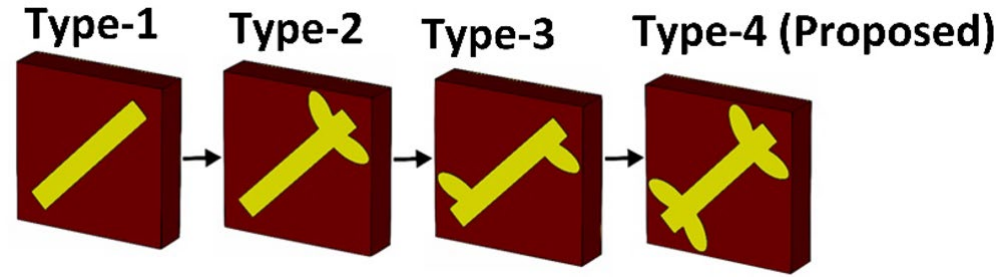
Optimization of the top dipole structure.

The top layer (dipole) of the metasurface is a 45° inclined metallic patch, which passes through four unique evolutionary stages as shown in Fig. 6:

Type-1: Simple microstrip.
Type-2: Microstrip with two leaf stubs connected to the top-right corner.
Type-3: Microstrip with a single leaf stub connected to the top-right and bottom-left corners.
Type-4: Proposed microstrip with two leaf stubs connected to the top-right and bottom-left corners.

**Figure 7:**
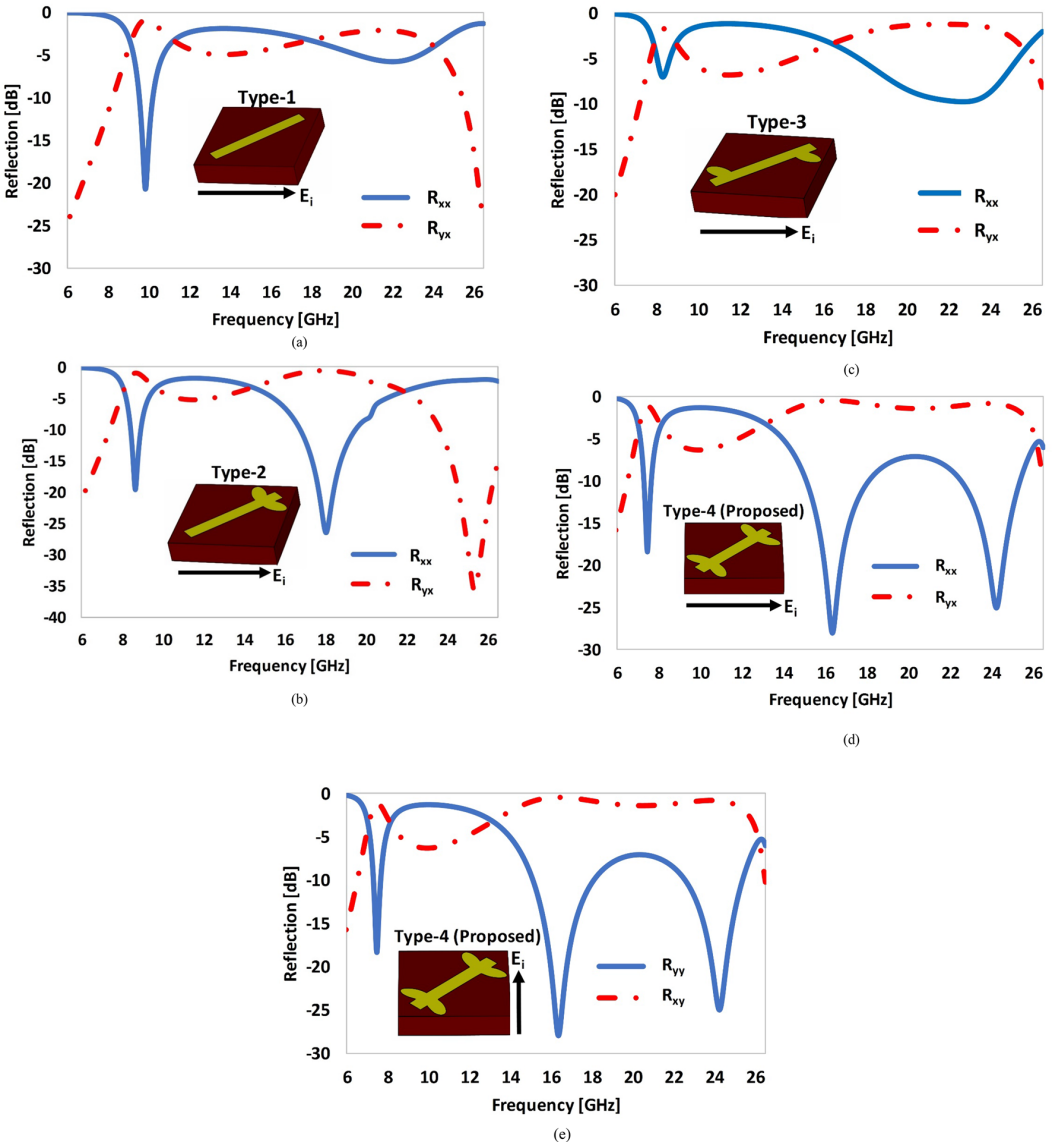
Comparison of the co- and cross-polarization reflection coefficients by varying the geometry of the dipole structure: **(a)** type-1, **(b)** type-2, **(c)** type-3, **(d)** type-4, **(e)** type-4 (y-polarized incident waves).

Initially, each of the metasurface of type-1 to type-4 have been exposed to *x*-polarized incident to investigate the polarization conversion phenomenon by analyzing the co (*R*_*xx*_) and cross (*R*_*yx*_) polarization reflection coefficients (Fig. 7). A threshold of *R*_*yx*_ ≥ − 3 dB has been set as a benchmark to define the purity and bandwidth of polarization conversion of the proposed metasurface. It can be clearly noticed that optimization of the dipole structure mainly controls the purity and associated bandwidth of polarization conversion of the metasurface in the upper resonant band (13–25 GHz). The lower resonant band is also shifted due to these design changes. For type-1 a dominant resonance is observed at 10 GHz, where the value of the cross-polarization reflection coefficient (*R*_*yx*_) is greater than − 3 dB in the lower resonant band (Fig. 7a). The polarization conversion is poor (i.e., *R*_*yx*_ ≤ − 3 dB) in the upper frequency band (17.5–26 GHz). Type-2 gives two dominant resonant bands with *R*_*yx*_ ≥ − 3 dB in the lower (8.5 GHz) and upper (15–18 GHz) frequency bands (Fig. 7b). The polarization conversion response of type-3 metasurface is significantly degraded relative to type-1 and 2 (Fig. 7c). The proposed metasurface (type-4) has two prominent polarization conversion bands with a *R*_*yx*_ ≥ − 3 dB, the first narrowband resonance covers 7–8 GHz frequency range, while second wideband resonance covers 13.3–25.5 GHz frequency range (Fig. 7d). The introduction of four elliptical shape leaves in type-4 causes the resonance at higher frequency due to the asymmetry of the elliptical leaves attach to the dipole. The proposed resonator supports, two orthogonal modes, named as “low mode” and “high mode”. The ‘low mode’ is the transverse magnetic (TM) mode, which is excited in the lower resonant bands (7.4 GHz and 16.4 GHz), and the ‘high mode’ is the transverse electric (TE) mode, excited in the upper resonant band (24. 2 GHz). An identical response has been observed due to the symmetry of the proposed structure for *y*-polarized incident waves (Fig. 7e). In general, the co-polarization coefficient “*R*_*xx*_” for the frequency range is lower than − 7 dB, while it reaches to the maximum of − 25 dB at 16 GHz and 24.5 GHz. The cross-polarization coefficient “*R*_*yx*_” in the frequency range from 13.3 to 25.5 GHz is higher than − 1.6 dB. The PCR in this frequency range is most equal to 1 with a fractional bandwidth of 62%.

**Figure 8:**
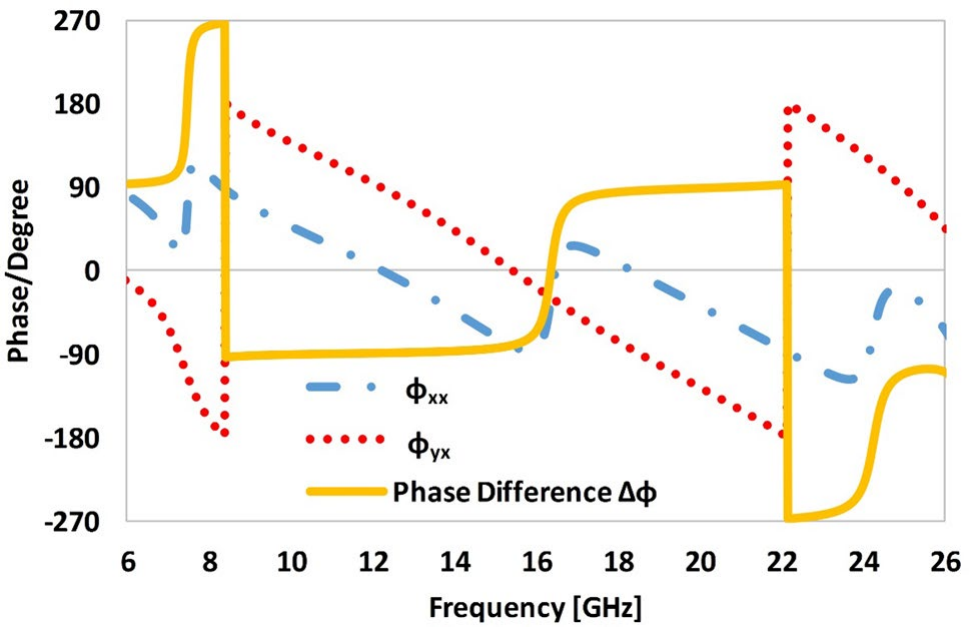
Reflection phase analysis of the proposed metasurface for *x*-polarized incident waves.

For the *x*-polarized incident wave, the phase difference ($\Delta \phi = \phi_{xx} - \phi_{yx}$) between the co- and cross-polarized reflected waves is shown in Fig. 8. It is evident from Fig. 7d that the magnitude of the co- and cross-polarization reflection coefficients attain identical values (*R*_*yx*_ = *R*_*xx*_) at four unique frequencies of 7 GHz, 8.1 GHz, 13.1 GHz and 26 GHz with a phase difference of $\Delta \emptyset = \pm 90^{ \circ }$ or $\pm 270^{ \circ }$ in these frequency bands (as shown in Fig. 8). Based on these findings it can be estimated that the reflected wave at these four frequencies is circularly polarized. In other words, the proposed metasurface has the ability to convert a linearly polarized incident wave into a circular polarized reflected wave of specific handedness. At 7 GHz, *R*_*xx*_ = *R*_*yx*_ and the phase difference (Δ*ϕ*) at this frequency is equal to + 90° (i.e., *x*-polarized component of the reflected wave is 90° ahead of the *y*-polarized counterpart), which show that the resultant reflected wave is left-hand circularly polarized (LHCP). For the frequencies of 8.1, 13.1 and 26 GHz, the phase difference (Δ*ϕ*) is equal to −90° or 270°. The magnitude of the co and cross-polarization reflection are  also equal in these bands. The phase difference of − 90° or 270° can lead to a right-hand circularly polarized (RHCP) reflected wave.

### Optimization of size (length × width) of the unit cell of the proposed metasurface

In this section, the reflection response of the proposed metasurface is investigated by scaling the size (length × width) of the unit cell. As discussed in the previous section, the unit cell of the proposed metasurface of Fig. 1 has a length and width both equal, i.e. *B* = 7 mm. When it was excited with waves of known polarization, the metasurface generates three resonant frequencies at which the co-polarization reflection coefficient is minimum and the cross-polarization coefficient is maximum. These resonant frequencies are *f* = 7.4 GHz, 16.4 GHz and 24.2 GHz, as shown in Fig. 7d,e.

**Figure 9:**
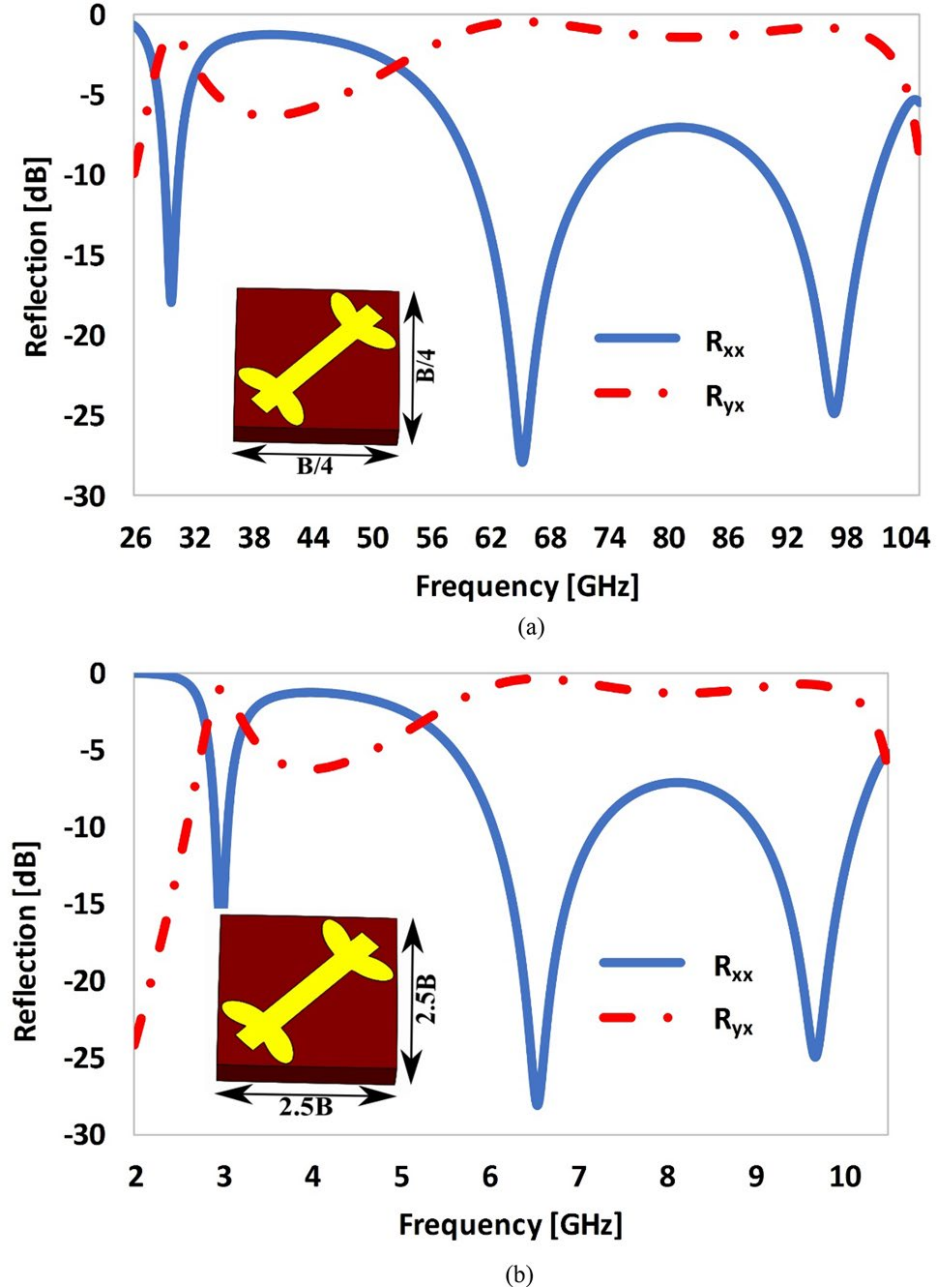
Reflection response of the proposed metasurface by scaling the size of the unit cell: **(a)** size = B/4 × B/4, **(b)** size = 2.5 B × 2.5 B (where B = 7 mm).

When the size of the unit cell is reduced to one quarter (i.e., size = *B*/4 × *B*/4), the collective load capacity of the unit cell is reduced and a proportional increase in the resonant frequency occurs as shown in Fig. 9a. Alternatively, when the size of the unit cell is downscaled by a factor of 4, the resonant frequencies of the original cell (i.e. 7. 4, 16. 4 and 24. 2 GHz), were increased by a factor of four, to 29 GHz, 65 GHz and 97 GHz, respectively. Similarly, when the size of the unit cell is scaled up to 2.5*B* × 2.5*B*, the load capacity of the structure has been raised, which results in a proportional decrease in the frequency response of reflection coefficient as depicted in Fig. 9b, i.e., *f* = 3 GHz, 6.5 GHz and 9.6 GHz. It is worth mentioning that the shape of the frequency response of the reflection coefficient remain unchanged irrespective of upscaling or downscaling the size of the unit cell. It is worth mentioning that the electromagnetic boundary conditions were kept identical for the original unit cell and its scaled counterparts. The simulation frequency sweep for the scaled or transformed versions of the metasurface unit cells have been carefully selected based on the inverse relationship between size of the cell under test and the frequency.

### Optimization of the length (L_p_) of the dipole structure

**Figure 10:**
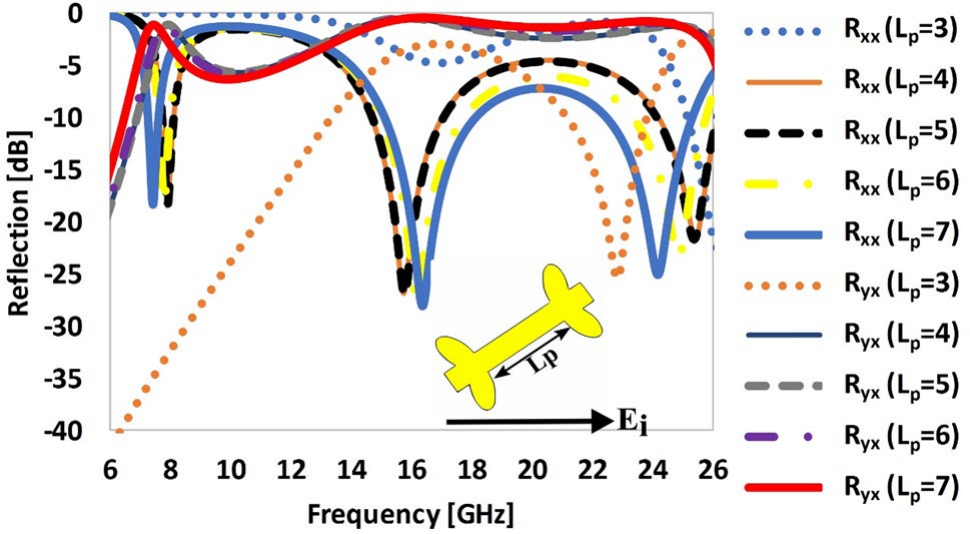
Reflection response of the proposed metasurface by varying the length of the patch (L_p_).

To further explore the design, we illustrate the response of the unit cell by changing the length (*L*_*p*_) of the dipole patch. For *x*-polarized incident waves, the co- and cross-polarization coefficients are analyzed by changing the length (*L*_*p*_) from 3 to 7 mm (Fig. 10). It can be seen from Fig. 10 that as the length of the patch decreases (from 7 to 3 mm) the co-polarization reflection coefficient (*R*_*xx*_) in the lower (7 GHz) and higher (24 GHz) resonant bands is shifted towards the higher frequency end, while the middle resonant band (16 GHz) is shifted toward lower frequencies. The frequency response of the cross-polarization reflection coefficient is slightly shifted toward higher frequencies when *L*_*p*_ is reduced from 7 to 3 mm. The response of surface for patch length of 3 mm is totally distorted, resulting in poor polarization conversion efficiency and reduced bandwidth than the patch of longer length.

### Optimization of the thickness (H) of the dielectric substrate

**Figure 11:**
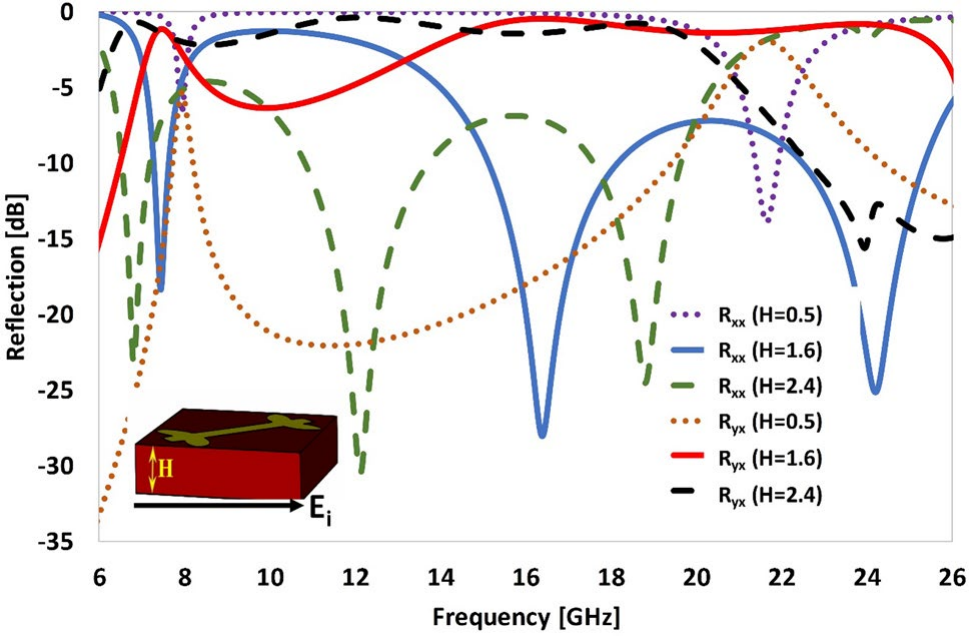
Reflection response of the proposed metasurface by varying the thickness of the substrate.

The bandwidth and degree of polarization conversion of the engineered metasurface is affected by altering the thickness of the dielectric substrate. It is clear from Fig. 11 that as the thickness of the substrate is increased from 0.5 to 2.4 mm, the bandwidth of polarization conversion is altered. The polarization conversion response is very poor for substrate thickness of 0.5 mm. However as the thickness is increased from 1.6 to 2.4 mm, the polarization conversion bandwidth gets expanded (17.47 GHz) and the three resonant frequencies of *R*_*xx*_ (*f*_*1*_ = 7.4, *f*_*2*_ = 16.4 and *f*_*3*_ = 24.2 GHz) are shifted downwards (to *f*_*1*_ = 6.5 GHz, *f*_*2*_ = 12 GHz and *f*_*3*_ = 18.5 GHz. This is due to the fact that as the substrate gets thicker, the effective inductance of the unit cell is increased. However, by increasing the thickness of the substrate beyond certain range, the design not only become bulkier in volume but also the performance of the metasurface degrades due to the surface wave losses in thicker substrates. Thus, in designing a polarization converter, performance, volume and fabrication complexities of the metasurface must be taken into consideration.

### Parametric extraction

**Figure 12:**
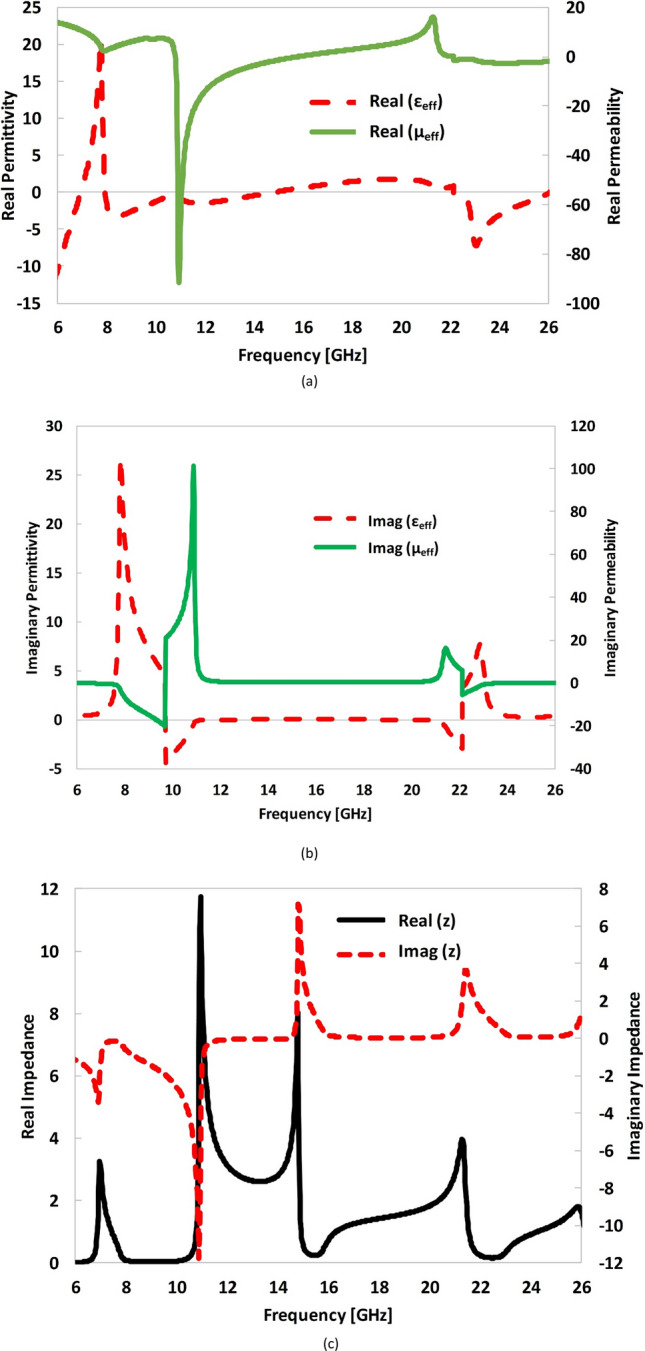
Extracted parameters of the metasurface **(a)** real components of *ε*_*eff*_, and *µ*_*eff*_ **(b)** imaginary components of *ε*_*eff*_, and *µ*_*eff*_ and **(c)** impedance of the metasurface.

The effective permittivity, effective permeability, effective impedance of the proposed design is extracted by *S*-parameter-retrieval method^46–48^ as shown in Fig. 12. The following expressions were employed to extract these intrinsic parameters of the metasurface:

5 $$z = \pm \sqrt {\frac{{\left( {1 + S_{11} } \right)^{2} - S_{21}^{2} }}{{\left( {1 - S_{11} } \right)^{2} - S_{21}^{2} }}}$$

6 $$e^{{jnk_{0} H}} = \frac{{S_{21} }}{{1 - S_{11} \frac{z - 1}{{z + 1}}}}$$

7 $$\varepsilon_{eff} = \frac{n}{z}$$

8 $$\mu_{eff} = n \times z$$

where *z*, *S*_*11*_, *S*_*21*_, are the normalized impedance, reflection coefficient and transmission coefficient, respectively. Likewise, *n* is the refractive index, *k*_*o*_ is the wave number and *H* is the thickness of the substrate. The effective permittivity, *ε*_*eff*_ and effective permeability, *µ*_*eff*_ are evaluated using (7) and (8), respectively.

It is observed that the extracted real and imaginary parts of the retrieved permittivity and permeability at three resonance frequencies of 7.4, 16. 4 and 24.2 GHz are nearly equal. This satisfy the condition of impedance matching of the medium (proposed surface) with the free space resulting in minimum co-polarization reflection and maximum PCR of the surface^49^. The retrieved effective parameters are listed in Table. 1.

**Table 1:** Extracted parameters of the proposed metasurface.

Frequency (GHz)	Permittivity (ε_eff_)		Permeability (μ_eff_)		Impedance (z)	
	Re (ε_eff_)	Im (ε_eff_)	Re (μ_eff_)	Im (μ_eff_)	Re (z)	Im (z)
7.4	7.591	1.680	7.827	− 0.585	1.1	− 0.15
16.4	0.810	0.019	0.885	0.177	1.05	0.09
24.2	− 2.618	0.318	− 2.639	− 0.070	0.98	0.07

## Conclusions

We have simulated and fabricated a thin, dual-band polarization conversion metasurface in the microwave frequency range. The metasurface converts incident waves of known linear polarization state (*x* or *y*) to the orthogonal or cross linear polarization state (*y* and *x*) in the reflection mode. This polarization conversion has been attained in two distinct frequency bands, i.e., a narrowband ranging from 7.1 GHz to 8 GHz with a PCR > 95% and a wideband ranging from 13.3 GHz to 25.8 GHz, with a PCR ~ 60–95%. Also, at four distinct frequencies, linear polarized wave can be converted into circular polarized wave at 7, 8.1, 13.1, and 26.2 GHz. The intrinsic parameters of the metasurface were retrieved using the S-parameter extraction method, and the values of permittivity and permeability were found nearly equal at the three resonant frequencies (7. 4 GHz, 16.2 GHz and 24. 2 GHz) which supports the argument of polarization convertion in the targeted frequency bands. The proposed metasurface should have great potential in sensor application, stealth technology, electromagnetic measurements, and antenna design because of its ultra-wideband, highly efficient and dual band performance.
